# From raw clinical data to robust prediction: an AI framework for early lymphedema detection

**DOI:** 10.1186/s12874-026-02805-4

**Published:** 2026-03-13

**Authors:** Ibrahim Sadek, Shafiq Ul Rehman, Ahmed Gehad, Esraa G. Eltasawi, Ahmed AbdelKader, Rawan Abdelnasser, Dina Nashaat, Raef Mourad Zaki, Lamees N. Mahmoud

**Affiliations:** 1 Biomedical Engineering Department, Faculty of Engineering, Helwan University, Helwan, Egypt; 2 College of Information Technology, Kingdom University, Riffa, Kingdom of Bahrain; 3 Biomedical Engineering Department, Faculty of Engineering, Cairo University, Cairo, Egypt; 4 Research Center, Baheya Center for Early Detection and Treatment of Breast Cancer, Giza, Egypt; 5 Department of Physical Therapy, Baheya Center for Early Detection and Treatment of Breast Cancer, Giza, Egypt

**Keywords:** Early detection, Lymphedema, Missing data, Machine learning, TabPFN, LightGBM

## Abstract

**Purpose:**

Breast cancer related lymphedema (BCRL) is a frequent postoperative complication that can substantially impair physical function and quality of life. While objective detection tools such as bioimpedance spectroscopy and imaging are accurate, they are resource intensive and difficult to deploy at scale in routine clinical workflows. Early identification of patients at elevated risk using routinely available clinical data may enable timely surveillance and intervention.

**Methods:**

We developed a comparative machine learning framework for postoperative BCRL risk prediction using routinely collected clinical data from 1,328 breast cancer patients treated at a single tertiary care center. Twenty four demographic, clinical, pathological, and treatment related variables were analyzed in tabular form. Nine classifiers spanning linear, ensemble, boosting, and transformer based models were systematically evaluated across seven preprocessing pipelines addressing missing data, feature scaling, class imbalance, and decision threshold optimization. Model performance was assessed using minority class F1 (F1_m), accuracy, and the Brier score to jointly evaluate discrimination and probabilistic calibration.

**Results:**

Predictive performance varied substantially across preprocessing strategies. When emphasis was placed on minority-class discrimination and probabilistic calibration, ensemble-based models including Random Forest, CatBoost, and LightGBM, as well as the transformer-based TabPFN, achieved the strongest results under the task-specific Custom preprocessing pipeline. Among these, TabPFN attained the highest overall performance, reaching F1_m values of approximately 0.86 and accuracy around 0.89 on the held-out test set. Random Forest, CatBoost, and LightGBM demonstrated robust and competitive performance, with F1_m values in the range of approximately 0.78 to 0.80 and accuracies around 0.83 to 0.86, together with stable Brier scores. In contrast, preprocessing pipelines that avoided targeted data exclusion yielded more conservative but consistent performance, highlighting trade-offs between discrimination, calibration, and data retention in real-world clinical settings.

**Conclusion:**

This study demonstrates that preprocessing choices play a critical role in BCRL risk modeling using real world clinical data. While task specific preprocessing can substantially improve minority class performance, such gains should be interpreted cautiously in light of data exclusion and potential selection bias. The proposed framework provides a transparent and reproducible foundation for retrospective BCRL risk stratification and offers a methodological template for future multi center validation studies in postoperative breast cancer care.

**Graphical Abstract:**

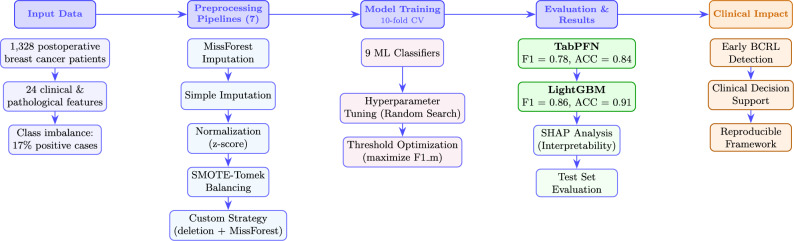

## Introduction

Lymphedema following breast cancer treatment is a clinically important, low-prevalence outcome whose consequences are largely irreversible if diagnosed late [1]. Our motivation for developing a rigorous, comparative methodology arose from three practical needs identified in this project: (1) the requirement to prioritize reliable predictive performance for a rare but clinically meaningful outcome, (2) the inherent challenges of real-world clinical data (substantial missingness, mixed data types, and severe class imbalance), and (3) the need to produce models that are accurate, robust, interpretable, and reproducible for downstream validation and clinical evaluation.

First, clinical model evaluation must extend beyond reporting a single best accuracy value. For imbalanced clinical tasks, the minority-class F1 score (F1_m) provides a succinct measure that balances precision and recall for the lymphedema class, whereas overall accuracy reflects global correctness across the dataset. To complement discrimination-based metrics, we additionally report the Brier score to assess probabilistic calibration. To address decision threshold sensitivity, threshold tuning was incorporated as a routine step during model training: rather than relying on the default decision cutoff, operating thresholds were swept within the training data and the value maximizing F1_m was selected for each candidate model, ensuring that the chosen operating point reflects the intended optimization objective rather than an arbitrary default.

Second, the clinical dataset exemplified common real-world challenges, including substantial feature-level missingness, mixed numerical and categorical variables, and a dominant majority class. Accordingly, preprocessing decisions substantially affect model inputs and thereby influence observed F1_m, accuracy, and Brier-based calibration performance. The Brier score was selected because it directly quantifies probabilistic accuracy and calibration, which is particularly important for imbalanced clinical prediction tasks where reliable risk estimation is required [2].

To mitigate fragile or over-optimistic results, our method systematically compares alternative missing-data strategies (MissForest, simple mean/mode imputation, and a targeted custom deletion followed by MissForest), balancing strategies where applicable, and a diverse set of model families (Logistic Regression, KNN, SVM, Random Forest, Extra Trees, LightGBM, CatBoost, XGBoost, and TabPFN). Presenting results across all pipeline–model combinations highlights which models are robust to preprocessing choices and which apparent gains are artifacts of a specific pipeline. Third, reproducible and transparent performance estimation is essential.

We enforce strict cross-validation practices by fitting all preprocessing steps within training folds and using stratified cross-validation for hyperparameter selection and evaluation. This approach prevents data leakage and yields reliable estimates of F1_m and accuracy variability across folds. The proposed methodology serves as a practical template for researchers working with clinical tabular data, particularly in the presence of missing values and class imbalance. Specifically, it enables researchers to identify robust model–preprocessing pairs, prioritize F1_m through per-model threshold selection with clear reporting of accuracy, and assess how imputation and balancing choices affect both discrimination and calibration. Finally, our approach emphasizes ethical and operational best practices, including transparent reporting of preprocessing decisions, performance variability, and computational settings, thereby allowing models to be validated, compared, and responsibly assessed prior to clinical deployment or prospective studies.

## Methods

In this section, we provide a comprehensive description of the methodological framework employed in our study to facilitate replication. The study, conducted in collaboration with Baheya Hospital (Baheya Foundation for Early Detection of Breast Cancer), Cairo, Egypt, aims to develop robust predictive models for postoperative lymphedema risk stratification in breast cancer patients, building on prior efforts [3, 4]. Lymphedema, characterized by chronic lymphatic fluid accumulation following lymph node dissection, represents a significant clinical challenge due to its progressive nature and the absence of curative treatments [1].

Early risk prediction using routinely collected postoperative data can enable proactive interventions and potentially mitigate long-term morbidity and improve quality of life; prior symptom-based studies have demonstrated the feasibility of logistic regression for modeling postoperative baseline indicators such as arm tightness and heaviness [5], which is cited to provide clinical motivation rather than to imply methodological equivalence. This study was approved by the Institutional Review Board (IRB) of the Baheya Foundation for Early Detection of Breast Cancer (IRB No: 202411270053). A total of 1,328 postoperative breast cancer patients comprised the study cohort, from which descriptive statistics of variables included in model development were derived (Table 1).

**Table 1:** Baseline demographic, clinical, and treatment characteristics of variables included in model development. BMI denotes body mass index; DM denotes diabetes mellitus; T, N, and M denote tumor size, nodal involvement, and distant metastasis, respectively; MRM denotes modified radical mastectomy; ALND denotes axillary lymph node dissection; SLNB denotes sentinel lymph node biopsy

Variable	n (%)/Mean ± SD
Age (years)	53.92 ± 11.79
Menopausal status	Postmenopausal: 563 (52.9%) Premenopausal: 501 (47.1%)
BMI (kg/m${}^2$)	32.68 ± 6.60
Past diabetes mellitus	No: 1032 (77.3%) Yes: 303 (22.7%)
Past hypertension	No: 860 (64.4%) Yes: 475 (35.6%)
Past cardiac disease	No: 1238 (92.7%) Yes: 97 (7.3%)
Laterality	Left: 608 (45.5%) Right: 594 (44.5%) Bilateral: 133 (10.0%)
T stage	T0–T1: 177 (18.6%) T2: 449 (47.4%) T3–T4: 318 (33.6%)
N stage	N0: 523 (55.2%) N1–N3: 424 (44.8%)
M stage	M0: 1320 (98.9%) M1: 15 (1.1%)
Specimen type	MRM: 773 (58.3%) Conservative: 528 (39.8%)
Number of lymph nodes removed	2.28 ± 3.76
Lymph node procedure	SLNB: 402 (81.5%) ALND: 68 (13.8%) Other: 23 (4.7%)
Peritumoral lymphovascular invasion	Present: 475 (49.7%) Absent: 475 (49.7%)
Chemotherapy	Yes: 840 (62.9%) No: 495 (37.1%)
Radiotherapy	Yes: 709 (53.1%) No: 626 (46.9%)
Hormonal therapy	Yes: 773 (57.9%) No: 562 (42.1%)
Lymphedema outcome	No: 1107 (82.9%) Yes: 228 (17.1%)

In this cohort, lymphedema diagnosis was clinically and physiotherapy led rather than imaging based. Diagnosis was established through structured clinical assessment conducted during postoperative follow up, including systematic history taking to identify lymphatic risk factors, onset and progression of swelling, and patient reported symptoms such as heaviness, tightness, discomfort, and diurnal variation. Physical examination included inspection and palpation to assess limb asymmetry, tissue consistency, and the presence of pitting or fibrotic changes. Objective limb measurements were performed using standardized anatomical landmarks and consistent measurement protocols to quantify side to side differences and monitor progression. Functional assessment, including evaluation of joint mobility, muscle strength, pain, and impact on daily activities, formed an integral component of diagnosis. Disease severity and progression were classified according to the International Society of Lymphology staging system. These procedures reflect real world clinical practice in physiotherapy led lymphedema management and are consistent with established guidelines [6, 7].

Patient data comprised a comprehensive set of demographics and clinicopathological variables, including age, body mass index (BMI), comorbidities (e.g., diabetes), tumor stage (TNM classification), lymph node involvement, surgical details, treatment modalities (e.g., chemotherapy and radiotherapy), and symptomatic indicators (e.g., swelling and pain). To ensure scientific integrity and reproducibility, all analyses were performed in Python, employing libraries such as scikit-learn for model development, imbalanced-learn for addressing class imbalance, and MissForest-based imputation for handling missing data, with model performance evaluated using F1_m, accuracy, and the Brier score to capture both discrimination and probabilistic calibration. The dataset exhibited a pronounced class imbalance (17% positive cases), a common challenge in clinical prediction that can bias models toward the majority class if unaddressed, as discussed in [8] for medical prediction tasks, including breast cancer–related outcomes.

### Data preparation and splitting strategy

To ensure robustness and reproducibility, the dataset was randomly shuffled prior to splitting to mitigate potential ordering effects arising from the original data collection process (e.g., chronological or demographic clustering), using a fixed random seed (42). The data were then partitioned into training (80%) and testing (20%) subsets with no overlap between sets, providing an unbiased hold-out test set for final performance assessment. All preprocessing, model fitting, threshold tuning, and hyperparameter optimization were conducted exclusively within the training data, thereby preventing information leakage and avoiding optimistic performance estimates. This split served as a stable foundation for subsequent cross-validation–based model development and evaluation.

Following shuffling, the dataset was stratified into an 80% training set (1,062 records) and a 20% hold-out test set (266 records). Stratification preserved the original class distribution (83% negative, 17% positive) in both subsets, ensuring that the test set accurately reflected the real-world imbalance. This split was performed prior to any preprocessing or modeling to fully isolate the test set as an unbiased benchmark for final evaluation. The training set was used exclusively for preprocessing strategy exploration, model training, hyperparameter tuning, and cross-validation, whereas the test set remained untouched until the final evaluation stage.

**Fig. 1:**
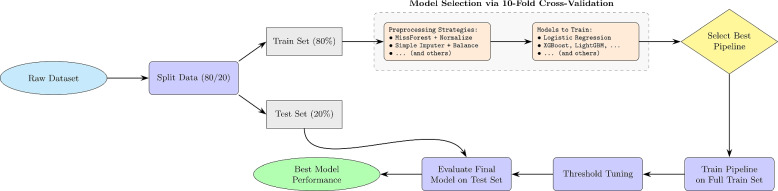
Pipeline illustrates data preprocessing, model selection, and evaluation for early lymphedema prediction

Figure 1 summarizes the proposed framework for lymphedema prediction, highlighting the sequence of data preprocessing, model selection, and evaluation. This strategy aligns with established machine learning best practices for simulating real-world deployment scenarios and avoiding inflated performance estimates. In particular, random shuffling mitigates potential biases arising from latent ordering in the dataset (e.g., chronological or site-specific clustering), while stratification preserves the underlying class distribution across splits. Together, these steps ensure a fair and representative partitioning of the data for robust model assessment.

### Preprocessing pipelines and cross-validation on training data

Within the 80% training set, we conducted a systematic comparison of seven preprocessing pipelines to address key data challenges, including substantial missingness (up to 63% in certain variables such as lymph node counts), feature-scale heterogeneity, and pronounced class imbalance. Each pipeline implemented distinct combinations of imputation, normalization, and resampling strategies, enabling an evidence-based assessment of how preprocessing choices influence downstream model performance under realistic clinical data conditions. All pipelines were evaluated using 10-fold stratified cross-validation applied exclusively to the training data. Within each fold, preprocessing steps were fitted only on the sub-training portion and then applied to the corresponding validation fold, ensuring strict separation between training and validation data. This fold-wise fitting strategy prevents information leakage from validation samples and provides reliable estimates of model discrimination and calibration across preprocessing configurations.

1. MissForest – Normalize – Balance: Missing values were imputed using MissForest, a non-parametric random forest–based algorithm that iteratively predicts missing entries from observed feature relationships [9]. Continuous features were subsequently normalized using z-score standardization to improve numerical stability and support distance-based models. Class imbalance was addressed using SMOTE–Tomek resampling applied within training folds only, where SMOTE generates synthetic minority samples and Tomek links remove ambiguous majority instances [8].
2. MissForest – Normalize: Identical imputation and normalization steps as above, without class balancing, to isolate the effect of resampling.
3. MissForest: MissForest-based imputation only, retaining the original feature scales and class distribution as a baseline comparison.
4. Simple – Normalize – Balance: Simple imputation (mean for continuous variables and mode for categorical variables), followed by z-score normalization and SMOTE–Tomek balancing within training folds.
5. Simple: Simple imputation only, representing minimal preprocessing.
6. Custom: Targeted deletion of training samples labeled as “no lymphedema” with $\ge$2 missing values, resulting in a reduced but more complete training subset. Remaining missing values were imputed using MissForest. This deletion rule was applied consistently across models; for TabPFN, no additional imputation was required beyond this step due to its native support for missing values.
7. No preprocessing: Raw data with encoding only (one-hot encoding for categorical variables and label encoding where appropriate), serving as a control condition without imputation, normalization, or resampling.

These pipelines were selected to explore practical trade-offs between model performance, robustness, and computational complexity. MissForest-based imputation is capable of modeling nonlinear feature relationships by leveraging correlations among available demographic and clinical variables (e.g., age and body mass index) and has been shown to outperform simpler approaches such as KNN-based imputation in terms of imputation accuracy on biological datasets [9]. Class balancing strategies aim to mitigate bias toward the majority class and can improve F1_m in imbalanced settings, but may also introduce synthetic variability that does not reflect true patient distributions, as discussed in the clinical machine learning literature [8]. In contrast, simple imputation methods are computationally efficient and transparent but may be less effective in capturing complex feature dependencies.

### Model training, hyperparameter tuning, and comparison

Hyperparameter tuning was performed using a randomized search strategy embedded within the cross-validation framework to systematically optimize model configurations while mitigating overfitting risks inherent in imbalanced clinical datasets. Random search was selected over exhaustive grid search due to its superior efficiency in high-dimensional hyperparameter spaces, as demonstrated by Bergstra and Bengio [10]. For each classifier, scikit-learn’s ParameterSampler was used to evaluate 50 randomly sampled hyperparameter configurations drawn from predefined distributions tailored to each algorithm’s architecture.

For gradient boosting models (LightGBM), hyperparameters were sampled from predefined distributions reflecting practical trade-offs between model capacity and regularization: num_leaves was drawn from randint(5, 40) to control tree complexity; subsample and colsample_bytree were sampled from uniform distributions over [0.6, 1.0] to introduce stochasticity and feature subsampling; min_child_samples was drawn from randint(5, 100) to regularize leaf creation; and reg_alpha and reg_lambda were sampled from Uniform(0.0, 1.0) to penalize model complexity, consistent with the LightGBM framework [11]. Analogous distributions were defined for other classifiers, including logarithmic sampling of the regularization parameter *C* for linear models, integer-valued neighborhoods for KNN, and depth and ensemble size parameters for tree-based methods. All hyperparameter sampling used a fixed random state for reproducibility, and evaluation during search employed stratified cross-validation folds within the training data, optimizing F1_m. Sampling ranges were initially broad to span diverse complexity and regularization regimes and were subsequently refined through iterative exploratory experimentation.

Preliminary searches across these broad hyperparameter ranges were assessed by examining the distribution of sampled configurations and their corresponding F1_m values obtained during cross-validation. Parameter regions associated with pronounced variance, instability across folds, or indications of overfitting (e.g., markedly higher training performance relative to validation performance) were subsequently pruned. The sampling ranges were then iteratively refined to focus computational effort on subspaces that consistently yielded stable and strong F1_m performance across preprocessing pipelines and random seeds. This progressive narrowing improved search efficiency by reducing unnecessary evaluations while decreasing the likelihood of selecting overly complex models that performed well only due to chance.

Fifty random hyperparameter configurations per classifier were adopted as a pragmatic compromise between exploratory coverage and computational constraints. Each configuration generated by the randomized sampling procedure was evaluated using stratified 10-fold cross-validation applied to the 80% training data, and the configuration achieving the highest mean out-of-fold F1_m was selected. For boosting-based algorithms, early stopping based on an internal validation split within the training data was additionally employed to prevent unnecessary training of poorly performing configurations. This randomized search strategy, rather than exhaustive grid search, enables efficient exploration of high-dimensional hyperparameter spaces under a fixed computational budget, consistent with established recommendations in the machine learning literature [10].

Within each cross-validation fold, model fitting was performed on the sub-training subset (9/10 of the training data), with performance evaluated on the held-out fold (1/10). For boosting-based models that natively support early stopping (LightGBM, CatBoost, and XGBoost), validation data were explicitly supplied during training via fold-specific evaluation sets, and built-in stopping criteria or callbacks were used to terminate training once performance plateaued. This procedure identified fold-specific optimal iteration counts (e.g., number of trees or boosting rounds), reducing the risk of both underfitting and overfitting. The observed iteration behavior across folds was used to inform stable final configurations for reproducibility. For non-boosting models (e.g., Random Forest and Logistic Regression), standard fitting without early stopping was employed. In all cases, predictions were generated on both sub-training and validation subsets to compute fold-wise performance metrics, which were subsequently averaged to obtain mean training and validation scores for each hyperparameter configuration.

The selection of optimal hyperparameters was guided by the mean validation F1_m, emphasizing accurate detection of the clinically critical lymphedema cases in this imbalanced setting (83% negative vs. 17% positive). Prioritizing F1_m during tuning reflects the need to balance precision, to limit false positives that may prompt unnecessary clinical follow-up, and recall, to maximize sensitivity for early risk identification, with the harmonic mean penalizing extreme trade-offs. Once optimal hyperparameters were identified for each model, the corresponding configuration and associated mean training and validation metrics were retained, with early-stopping behavior for boosting models incorporated to inform stable final parameter settings.

Models were subsequently compared across preprocessing pipelines using their tuned configurations, consistent with prior evaluations of lymphedema risk prediction using ensemble and boosting approaches [4, 8], with performance aggregated across cross-validation folds. Evaluation focused primarily on F1_m, which directly quantifies the model’s ability to identify lymphedema cases in the presence of pronounced class imbalance, alongside accuracy as a complementary global performance measure. To assess probabilistic calibration and overall prediction reliability, we additionally report the Brier score, which captures the mean squared error of predicted probabilities and is widely recommended for clinical risk prediction tasks. Standard clinical performance indicators—including area under the ROC curve (AUC), sensitivity, specificity, and precision—were also computed to facilitate clinical interpretation of discrimination performance and error trade-offs, but were not used as primary criteria for model selection. Additional F1 variants (macro-averaged F1_Av and weighted F1_w as implemented by standard software libraries) were calculated for completeness only. Accuracy alone was not emphasized due to its susceptibility to class imbalance; for example, a trivial classifier predicting only the majority class would achieve approximately 83% accuracy while yielding an F1_m of zero, representing a complete failure to detect clinically relevant lymphedema cases. This metric hierarchy supported robust comparison across preprocessing strategies, with all results systematically logged to ensure transparency, reproducibility, and traceability.

### Final retraining, threshold tuning, and test evaluation

After identifying promising model–pipeline pairs via cross-validation, each model was retrained with optimized hyperparameters on the full 80% training set to maximize data utilization. Decision thresholds were subsequently tuned by sweeping probability cutoffs from 0.00 to 0.99 in increments of 0.01 and selecting the value that maximized F1_m on the training data. Final evaluation was then performed on the untouched 20% hold-out test set using these tuned thresholds.

This sequence—cross-validation on the training set, retraining on the full training data, threshold tuning, and final test evaluation—ensured that no information from the test set influenced preprocessing, hyperparameter selection, or threshold optimization. The resulting test-set performance reflected strong generalization, with boosting-based models such as LightGBM and CatBoost achieving F1_m values around 0.78 and accuracies around 0.83 under the Custom pipeline, and the transformer-based TabPFN model achieving the highest overall performance (F1_m $\approx$ 0.86, accuracy $\approx$ 0.89). These findings are consistent with the established effectiveness of gradient boosting methods [11, 12] and recent transformer-based approaches for tabular clinical data [13].

### Interpretability, robustness, and reproducibility

**Fig. 2:**
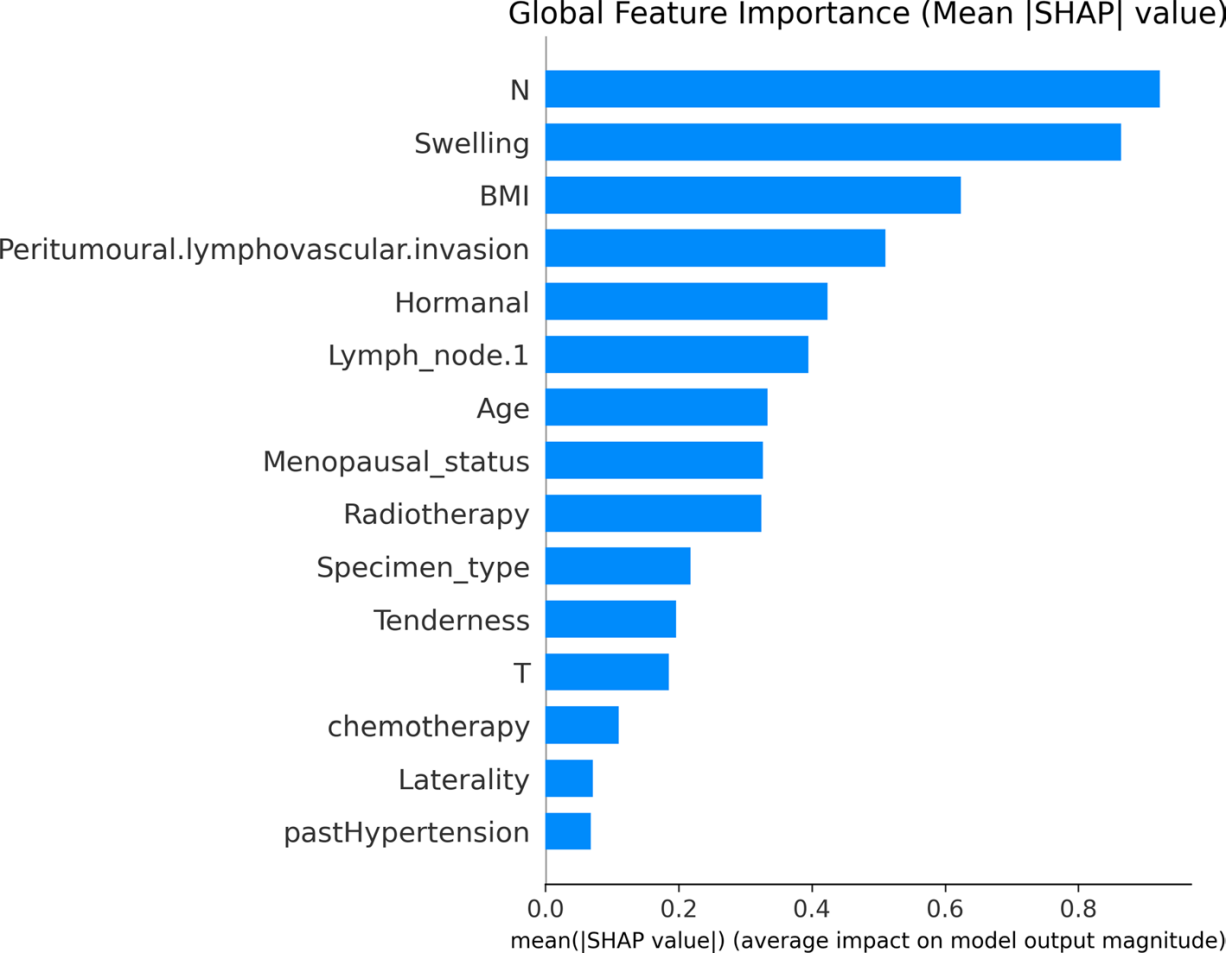
Cross validated SHAP feature importance for CatBoost under the Custom preprocessing pipeline. SHAP values were computed on validation folds only and aggregated across 10 folds, highlighting clinically plausible predictors of lymphedema risk

**Fig. 3:**
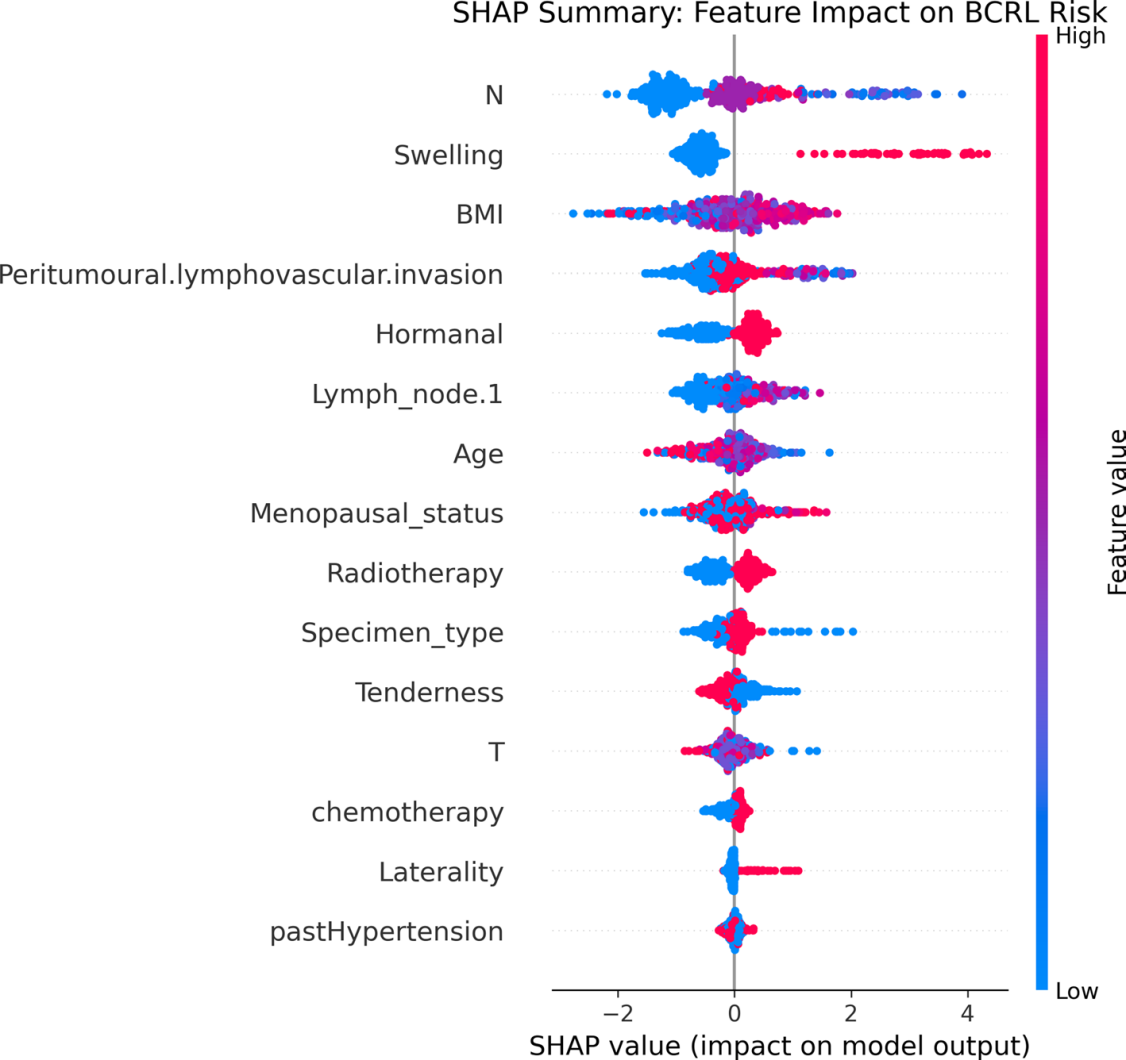
SHAP beeswarm plot illustrating the direction and magnitude of feature effects for CatBoost predictions under the Custom preprocessing pipeline. Each point represents a validation sample from cross validation folds

To enhance trust and clinical interpretability, model reliability was assessed using calibration-aware evaluation through the Brier score, complemented by discrimination metrics reported across preprocessing pipelines (Table 2). As an additional validation of model interpretability under the proposed preprocessing framework, we conducted a focused analysis using CatBoost combined with the Custom preprocessing pipeline. CatBoost was selected due to its strong performance among ensemble methods and its native support for tree based feature attribution. A stratified 10 fold cross validation procedure was applied, and SHAP values were computed exclusively on the held out validation fold at each iteration to ensure explanations were derived from unseen data. SHAP values were then aggregated across folds to obtain stable global importance estimates while avoiding optimistic bias (Figs. 2 and 3).

**Table 2:** Classification performance across preprocessing pipelines using complementary metrics. Results are reported as F1_m, accuracy (Acc), and Brier score (lower is better)

Model	Metric	MF+N+B	MF+N	MF	S+N+B	S	Custom	None
Logistic Regression	F1_m	0.51	0.52	0.52	0.51	0.52	0.69	–
	Acc	0.72	0.74	0.74	0.73	0.73	0.76	–
	Brier	0.19	0.19	0.19	0.19	0.19	0.19	–
KNN	F1_m	0.41	0.37	0.33	0.41	0.35	0.63	–
	Acc	0.67	0.64	0.58	0.67	0.60	0.69	–
	Brier	0.19	0.15	0.17	0.18	0.16	0.21	–
SVM	F1_m	0.49	0.53	0.46	0.47	0.49	0.68	–
	Acc	0.73	0.74	0.70	0.72	0.70	0.74	–
	Brier	0.16	0.12	0.13	0.20	0.12	0.20	–
Random Forest	F1_m	0.53	0.54	0.55	0.56	0.57	0.79	–
	Acc	0.75	0.75	0.75	0.75	0.76	0.86	–
	Brier	0.13	0.12	0.12	0.12	0.13	0.15	–
ExtraTrees	F1_m	0.53	0.52	0.52	0.55	0.55	0.74	–
	Acc	0.75	0.76	0.76	0.77	0.77	0.82	–
	Brier	0.13	0.15	0.14	0.14	0.15	0.17	–
LightGBM	F1_m	0.52	0.53	0.52	0.55	0.50	0.78	–
	Acc	0.73	0.72	0.72	0.73	0.71	0.84	–
	Brier	0.13	0.12	0.12	0.12	0.13	0.14	–
CatBoost	F1_m	0.53	0.53	0.53	0.52	0.54	0.79	–
	Acc	0.76	0.73	0.73	0.74	0.76	0.87	–
	Brier	0.12	0.14	0.14	0.12	0.14	0.14	–
XGBoost	F1_m	0.53	0.51	0.51	0.52	0.53	0.81	–
	Acc	0.74	0.72	0.72	0.74	0.74	0.86	–
	Brier	0.12	0.12	0.12	0.12	0.11	0.14	–
TabPFN	F1_m	–	–	–	–	–	0.86	0.59
	Acc	–	–	–	–	–	0.91	0.78
	Brier	–	–	–	–	–	0.11	0.11

Comparative analyses highlighted differing robustness profiles across models: transformer-based TabPFN exhibited sensitivity to explicit imputation strategies, with relatively lower F1_m under MissForest-based preprocessing, whereas gradient boosting models such as LightGBM demonstrated greater stability across imputation and balancing configurations. Reproducibility was ensured through fully scripted pipelines with fixed random seeds and serialized outputs, with code made available via a public repository upon request. Overall, the proposed methodology advances postoperative lymphedema risk prediction while providing a practical blueprint for addressing class imbalance, missing data, and evaluation rigor in clinical tabular modeling, extending prior symptom-driven and ensemble-based approaches [1, 4].

Preprocessing strategies in Table 2 are defined as follows: MF+N+B denotes MissForest-based imputation with feature normalization and class balancing; MF+N denotes MissForest-based imputation with normalization only; MF denotes MissForest-based imputation without normalization or balancing; S+N+B denotes simple imputation with normalization and class balancing; S denotes simple imputation only; Custom denotes the proposed task-specific preprocessing pipeline; and None denotes no preprocessing beyond basic encoding. Dashes (–) indicate model–pipeline combinations that were not evaluated, and bold values highlight the highest F1_m achieved by each classifier across preprocessing pipelines.

## Results and discussion

The results demonstrate the value of the proposed methodology by illustrating how preprocessing pipelines and machine learning classifiers interact to produce robust predictions despite real-world challenges such as high feature missingness (up to 63%) and severe class imbalance (83% negative vs. 17% positive). Model performance was primarily assessed using F1_m, Acc, and the Brier score to jointly evaluate discrimination and probabilistic calibration. These metrics were estimated via nested stratified 10-fold cross-validation on the 80% training split, followed by retraining and final evaluation on an untouched 20% hold-out test set after hyperparameter optimization and threshold tuning.

Table 2 summarizes the primary performance results across preprocessing pipelines using F1_m, accuracy, and Brier score, while Table 3 reports complementary clinical performance indicators, including sensitivity, specificity, precision, and area under the ROC curve (AUC). Blank cells indicate model–pipeline combinations that were not evaluated due to incompatibility or computational constraints. Together, these two tables provide a comprehensive and clinically interpretable comparison of model behavior across preprocessing strategies. When emphasis is placed on F1_m together with the Brier score, transformer-based TabPFN and ensemble methods, e.g., Random Forest and CatBoost achieve comparatively strong performance under the Custom preprocessing pipeline, although these gains should be interpreted cautiously given the substantial data exclusion and potential selection bias inherent to this strategy, which may limit generalizability to routine clinical settings where diagnoses are made under incomplete and heterogeneous data conditions.

**Table 3:** Supplementary performance metrics across preprocessing pipelines. Results are reported as sensitivity (Sens), specificity (Spec), precision (Prec), and area under the ROC curve (AUC). Preprocessing abbreviations follow Table 2

Model	Metric	MF+N+B	MF+N	MF	S+N+B	S	Custom	None
Logistic Regression	Sens	0.48	0.55	0.54	0.52	0.52	0.79	–
	Spec	0.91	0.86	0.87	0.88	0.88	0.67	–
	Prec	0.61	0.57	0.57	0.60	0.59	0.63	–
	AUC	0.72	0.74	0.74	0.73	0.73	0.76	–
KNN	Sens	0.60	0.76	0.69	0.59	0.65	0.81	–
	Spec	0.67	0.48	0.45	0.72	0.53	0.49	–
	Prec	0.39	0.26	0.25	0.36	0.27	0.55	–
	AUC	0.67	0.64	0.58	0.67	0.60	0.69	–
SVM	Sens	0.53	0.51	0.52	0.64	0.55	0.80	–
	Spec	0.86	0.91	0.83	0.75	0.84	0.62	–
	Prec	0.56	0.60	0.47	0.40	0.56	0.61	–
	AUC	0.73	0.74	0.70	0.72	0.70	0.74	–
Random Forest	Sens	0.49	0.48	0.48	0.49	0.51	0.74	–
	Spec	0.92	0.94	0.94	0.95	0.94	0.91	–
	Prec	0.60	0.68	0.68	0.68	0.66	0.86	–
	AUC	0.75	0.75	0.75	0.75	0.76	0.86	–
ExtraTrees	Sens	0.53	0.54	0.56	0.54	0.54	0.73	–
	Spec	0.90	0.89	0.87	0.91	0.89	0.82	–
	Prec	0.58	0.59	0.55	0.61	0.61	0.76	–
	AUC	0.75	0.76	0.76	0.77	0.77	0.82	–
LightGBM	Sens	0.50	0.48	0.44	0.50	0.44	0.77	–
	Spec	0.90	0.93	0.95	0.97	0.95	0.86	–
	Prec	0.58	0.66	0.67	0.77	0.60	0.80	–
	AUC	0.73	0.72	0.72	0.73	0.71	0.84	–
CatBoost	Sens	0.53	0.45	0.45	0.49	0.55	0.77	–
	Spec	0.90	0.95	0.95	0.95	0.90	0.89	–
	Prec	0.58	0.68	0.68	0.62	0.58	0.83	–
	AUC	0.76	0.73	0.73	0.74	0.76	0.87	–
XGBoost	Sens	0.55	0.49	0.49	0.54	0.54	0.78	–
	Spec	0.89	0.91	0.91	0.91	0.90	0.90	–
	Prec	0.56	0.60	0.60	0.59	0.58	0.85	–
	AUC	0.74	0.72	0.72	0.74	0.74	0.86	–
TabPFN	Sens	–	–	–	–	–	0.84	0.59
	Spec	–	–	–	–	–	0.92	0.91
	Prec	–	–	–	–	–	0.88	0.67
	AUC	–	–	–	–	–	0.91	0.78

To further support interpretability, CatBoost evaluated under the Custom preprocessing pipeline was used as a representative ensemble model for feature attribution analysis. Cross-validated SHAP analysis using the CatBoost model under the Custom preprocessing pipeline consistently highlighted lymph node involvement, the number of excised lymph nodes, exposure to radiotherapy, body mass index, and tumor stage as the most influential predictors of model output. These variables demonstrated stable importance across validation folds and are concordant with established clinical risk factors for breast cancer related lymphedema, supporting the clinical plausibility of the learned decision patterns. Figures 2 and 3 summarize the global and instance level explanations, respectively.

**Fig. 4:**
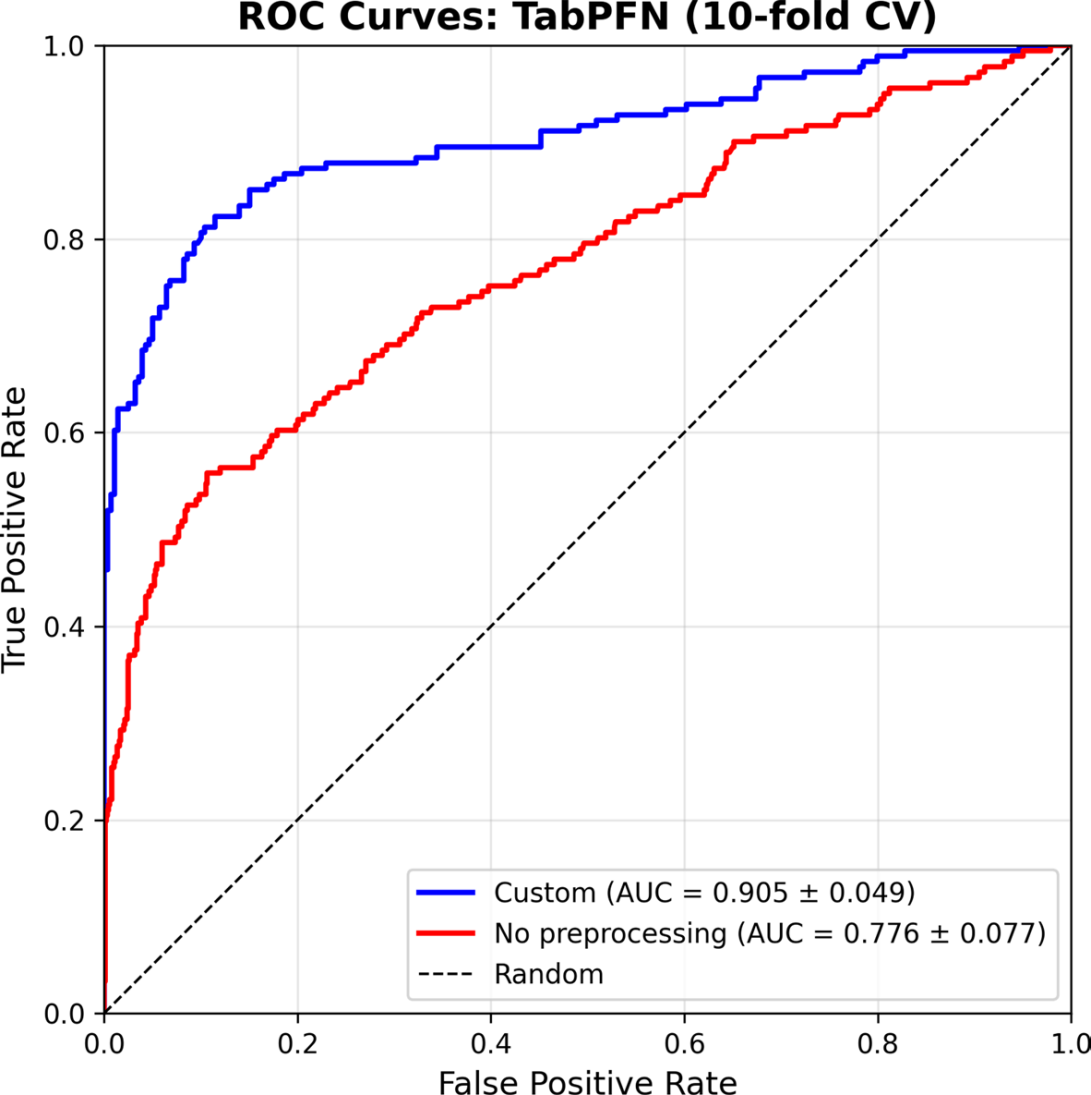
Receiver operating characteristic (ROC) curves for the TabPFN model under the Custom preprocessing pipeline and under No preprocessing, illustrating threshold-dependent trade-offs between sensitivity and specificity. The curves complement the AUC values reported in Table 3

Excluding the Custom pipeline, MissForest-based preprocessing without case deletion (with or without normalization) provided a stable and clinically realistic alternative, with ensemble models such as Random Forest, ExtraTrees, LightGBM, CatBoost, and XGBoost consistently achieving minority-class performance in the range F1_m $\approx$ 0.52–0.56, AUC $\approx$ 0.72–0.76, and Brier scores $\approx$ 0.12–0.15, indicating reasonable discrimination and calibration while retaining the full cohort and avoiding selection bias from aggressive data filtering. Given the comparatively strong discrimination achieved by TabPFN under the Custom and No-preprocessing pipelines, we additionally examined its receiver operating characteristic (ROC) curve to visualize sensitivity–specificity trade-offs across decision thresholds, complementing the summary AUC values reported in Table 3. Figure 4 shows ROC curves for the TabPFN model under the Custom preprocessing pipeline and under No preprocessing.

Across all evaluated models and preprocessing pipelines, specificity was consistently higher than sensitivity. This behavior reflects the severe class imbalance in the dataset (83% negative vs. 17% positive) and the choice to optimize decision thresholds using the minority-class F1 score (F1_m), which balances precision and recall rather than maximizing sensitivity alone. As a result, models favored conservative operating points that maintained low false-positive rates while still achieving meaningful detection of lymphedema cases. This trade-off is clinically plausible in postoperative risk stratification settings, where excessive false positives may lead to unnecessary follow-up procedures, while calibration-aware performance remains essential. Importantly, the observed specificity–sensitivity relationship is consistent with the reported F1_m and Brier scores and does not indicate degraded minority-class performance, but rather reflects deliberate threshold optimization under real-world data constraints.

As a sensitivity analysis addressing concerns regarding missing data mechanisms, we implemented multiple imputation by chained equations using scikit-learn’s IterativeImputer with a Bayesian ridge regression estimator. Bayesian ridge was selected as a stable default for chained-equation style regression, and posterior sampling was enabled to generate stochastic imputations. The imputation procedure used max_iter equal to 10, sample_posterior set to True, and random_state equal to 42. Five independently imputed datasets were generated, and performance was summarized by averaging results across imputations. This sensitivity experiment was conducted using LightGBM, one of the strongest and most stable models in the primary analysis, and followed the same training data partitioning strategy, with the Custom training filter applied prior to imputation. Preprocessing consisted of ordinal encoding, lymph node one hot expansion, MICE based imputation, and numeric normalization, followed by stratified ten fold cross validation with decision threshold tuning to maximize F1_m. Across imputations, the MICE LightGBM configuration achieved an average F1_m of approximately 0.74. While this performance exceeded that of simple imputation strategies, it remained inferior to the LightGBM model trained under the Custom preprocessing pipeline without multiple imputation, which achieved F1_m values around 0.78. These findings suggest that although probabilistic multiple imputation provides a principled robustness check against reliance on a single imputation method, performance gains in this dataset primarily arise from task specific data curation rather than from imputation alone. As expected, multiple imputation does not eliminate potential bias under fully missing not at random mechanisms, but it supports the stability of the main conclusions under alternative missing data assumptions.

### Preprocessing pipelines: impact on model performance

The seven preprocessing pipelines span minimal intervention (“No preprocessing”) through imputation- and balancing-focused strategies, enabling isolation of effects from imputation quality (MissForest-like vs. simple), normalization, and SMOTETomek resampling. The Custom pipeline applies targeted deletion of negative cases with $\ge$2 missing fields within the training split (reducing the effective training size) followed by model-specific preprocessing; for TabPFN, Custom and No preprocessing are evaluated using encoding-only (no explicit imputation). Under Custom, ensemble/boosting models achieve the strongest discrimination, e.g., LightGBM (F1_m$\approx$0.78, Acc$\approx$0.83, Brier$\approx$0.14) and XGBoost (F1_m$\approx$0.81, Acc$\approx$0.85, Brier$\approx$0.14), while simpler models also improve (KNN F1_m$\approx$0.63; SVM F1_m$\approx$0.68). However, these gains should be interpreted cautiously because Custom excludes a substantial fraction of cases and may introduce selection bias, which can limit generalizability to routine clinical settings with incomplete data.

### Model families: robustness across pipelines

 

- Linear and distance-based models (Logistic Regression, KNN, SVM): These models exhibited strong sensitivity to preprocessing choices, with their highest minority-class performance typically observed under the Custom pipeline, while performance under other pipelines remained modest (F1_m $\approx$ 0.35–0.55).
- Tree ensemble models (Random Forest, Extra Trees): These classifiers delivered comparatively stable performance across preprocessing strategies, achieving moderate-to-strong minority-class performance (F1_m $\approx$ 0.52–0.74) with consistent accuracy (Acc $\approx$ 0.75–0.82), indicating robustness to missing-data handling and class imbalance.
- Boosting-based models (LightGBM, CatBoost, XGBoost): Boosting models consistently achieved strong and stable results across most preprocessing pipelines, with improved minority-class discrimination under the Custom pipeline (F1_m up to $\approx$ 0.78–0.81) while maintaining reasonable calibration (Brier $\approx$ 0.12–0.15).
- Transformer-based TabPFN: TabPFN achieved its strongest performance when applied to minimally processed data (encoding only), particularly under the Custom and No preprocessing pipelines, but showed sensitivity to explicit imputation strategies, underscoring the importance of careful data handling when deploying transformer-based models on clinical tabular data.

**Fig. 5:**
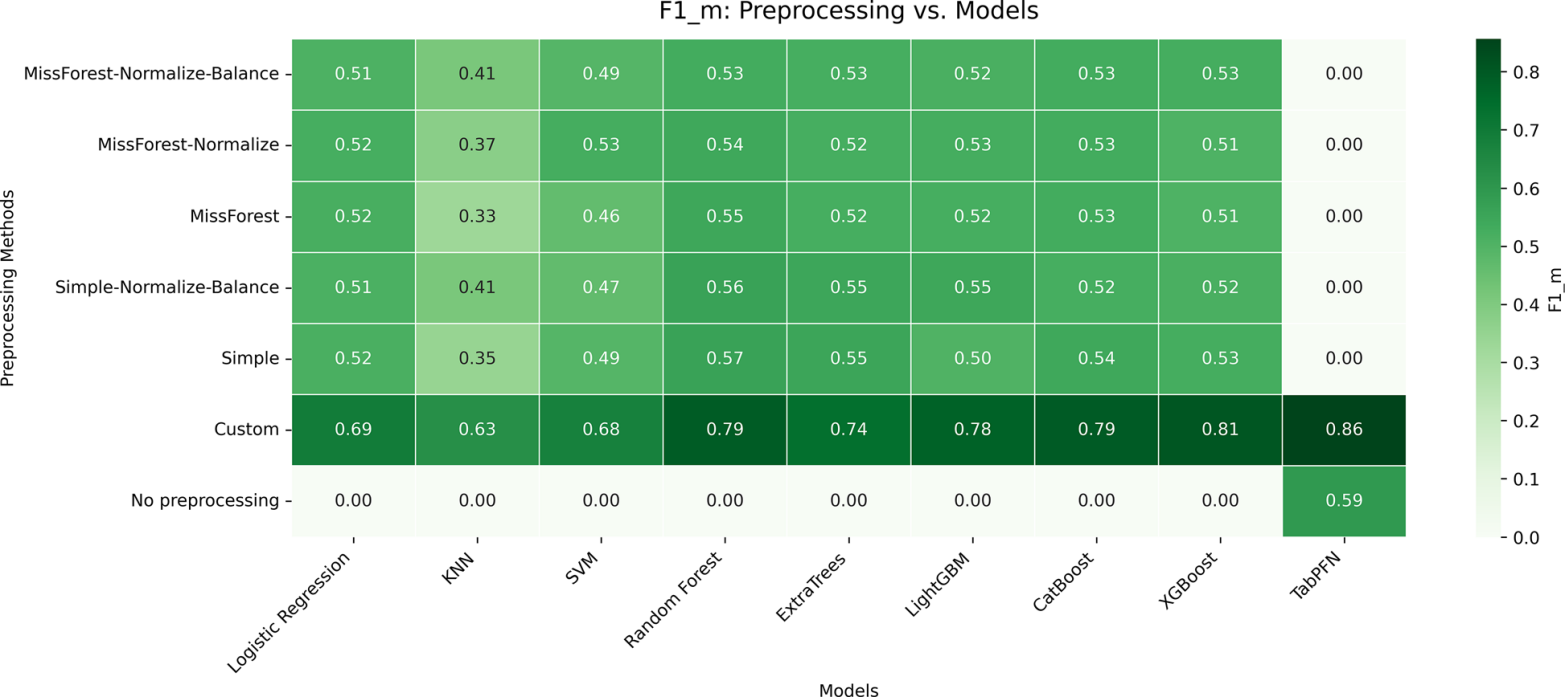
Heatmap of F1_m scores showing classifier performance across different preprocessing strategies

Figure 5 illustrates the relationship between model performance and preprocessing methods using the F1_m, enabling direct comparison of classifiers across different data-handling strategies. Together with the accompanying tables, these results highlight the methodological reproducibility of the proposed framework: researchers can adapt the evaluated preprocessing pipelines (e.g., using standard scikit-learn and imbalanced-learn components) to similar clinical prediction tasks while explicitly prioritizing minority-class performance and calibration under cross-validation. This approach not only advances breast-cancer-related lymphedema risk prediction but also provides a transparent and reproducible workflow for modeling imbalanced and incomplete clinical data, supporting responsible methodological evaluation in retrospective clinical research.

## Limitations

 

- Dataset specificity and generalizability: The proposed framework was developed and validated exclusively on a single-center clinical dataset (1,328 postoperative breast cancer patients from Cairo, Egypt), reflecting region-specific demographic and clinical practice patterns. Model performance may not generalize to multi-center, multi-ethnic, or international cohorts, where differences in surgical protocols, follow-up practices, socioeconomic factors, or unmeasured environmental influences may alter predictor–outcome relationships.
- Limited sample size and severe class imbalance: Despite the overall cohort size, only 228 patients (17%) exhibited lymphedema, resulting in a markedly imbalanced outcome distribution. This imbalance reduces statistical power for minority-class estimation, increases variance in cross-validation metrics such as F1${}_m$, and heightens susceptibility to overfitting. Model stability and performance may further degrade in datasets with fewer positive cases (e.g., fewer than 50 events), limiting applicability in smaller or early-stage cohorts.
- Assumptions in missing data handling: Advanced imputation methods such as MissForest implicitly assume missing-at-random (MAR) mechanisms, whereas real-world clinical data frequently exhibit missing-not-at-random (MNAR) patterns (e.g., lymph node counts absent in patients undergoing less extensive surgery). Such violations may introduce systematic bias into imputed values. The Custom preprocessing pipeline, which excludes negative cases with $\ge 2$ missing variables, improves internal data consistency but introduces potential selection bias by disproportionately retaining positive cases. This strategy may be unsuitable in datasets with uniformly high missingness ($>70\%$) or weakly structured missingness patterns, where MissForest convergence and downstream model reliability may be compromised.
- Computational and resource constraints: Several components of the framework, including MissForest (iterative random forest–based imputation) and TabPFN (transformer-based prior-data learning), are computationally demanding. Comprehensive cross-validation with hyperparameter tuning (50 random configurations per model–pipeline pair) can require substantial CPU or GPU resources, limiting feasibility in resource-constrained settings. Scaling the framework to substantially larger datasets (e.g., $>10{,}000$ patients) may necessitate parallelization or simplified preprocessing, and the limited interpretability of transformer-based models such as TabPFN may restrict adoption in clinical environments requiring transparent decision support.

## Conclusion

This study presented a rigorous comparative machine learning framework for the early prediction of breast cancer–related lymphedema (BCRL) using routinely collected postoperative clinical data. By systematically evaluating nine classifiers across seven preprocessing pipelines, the analysis demonstrated that preprocessing choices including missing-value handling, class balancing, and decision-threshold optimization substantially influence predictive performance in imbalanced clinical settings. When emphasis was placed on minority-class performance and probabilistic calibration, the transformer-based TabPFN and ensemble-based models, particularly Random Forest, CatBoost and LightGBM, achieved the strongest results under the Custom preprocessing pipeline. TabPFN attained the highest overall performance, reaching F1_m values of approximately 0.86 with accuracy around 0.89 and favorable Brier scores, while Random Forest and LightGBM also demonstrated strong and competitive performance (F1_m approximately 0.79 and 0.78, with accuracies around 0.85 and 0.83, respectively). These findings highlight the impact of task-specific preprocessing on discrimination and calibration performance, particularly for rare but clinically meaningful outcomes. Beyond predictive performance, the proposed framework emphasizes reproducibility and methodological transparency through strict separation of training and test data, nested cross-validation, and explicit threshold optimization based on F1_m.

Model reliability was further assessed using calibration-aware evaluation via the Brier score, providing complementary insight into probabilistic accuracy beyond discrimination metrics alone. Influential predictors were summarized using model intrinsic importance measures and clinical reasoning, which helped reduce the risk of misleading interpretations in high dimensional encoded feature spaces. Nevertheless, the observed performance gains particularly under the Custom pipeline should be interpreted cautiously. This preprocessing strategy involves targeted exclusion of incomplete negative cases, which improves internal data consistency but introduces potential selection bias and may limit generalizability to real-world clinical environments characterized by heterogeneous and incomplete data. In addition, the single-center nature of the cohort and the limited number of positive cases constrain external validity. In conclusion, this work provides a transparent and reproducible methodological framework for retrospective BCRL risk modeling, illustrating how preprocessing strategies, evaluation metrics, and model choice interact under real-world data constraints. While not intended for immediate clinical deployment, the framework offers a practical foundation for future multi-center validation studies and the development of clinically reliable machine learning models in postoperative breast cancer care.

## Data Availability

The dataset used in this study contains sensitive patient information and cannot be made publicly available. De-identified data may be shared upon reasonable request to the corresponding author and subject to institutional approval.

## Code availability

All custom code developed for data preprocessing, modeling, and evaluation is available from the corresponding author upon reasonable request.
